# Assessing the Feasibility of Partnering with a Home Visiting Program for Early Childhood Obesity Prevention

**DOI:** 10.1007/s10995-023-03780-8

**Published:** 2023-10-17

**Authors:** Julie M. Kapp, Brian Hall, Allison Kemner

**Affiliations:** 1 College of Health Sciences, University of Missouri, 806 Lewis Hall, Columbia, MO 65211 USA; 2 Parents as Teachers National Center, Saint Louis, MO USA

**Keywords:** Obesity, Home visit, Early childhood, Overweight, Adverse childhood experiences

## Abstract

**Purpose:**

Little empirical data exists evaluating the feasibility of partnering with established home visiting programs to implement early childhood obesity prevention programs, despite the recommendation to do so. To inform this gap, we evaluated the feasibility of collecting anthropometric measurements of children by home visitors across multiple sites, and the alignment of these measurements with children in need, including with adverse family experiences (AFEs) given emerging evidence suggests an association with childhood obesity.

**Description:**

Our proof-of-concept study included primary data collection of child anthropometric measurements through an established home visiting program in four states. This sample included 248 children ages 6 months to 5 years.

**Assessment:**

In the sample, 37.1% of the children had overweight or obesity, 50% were female, 64.2% Hispanic/Latinx, 15.8% non-Hispanic Black, and 42.3% from rural/small towns. Households included substantial needs: 87.1% were low income, 73.8% low education, and 59.3% underemployment. Regarding AFEs, 38.3% of the children had at least one, with the most common being mothers who were treated violently. A multivariable model revealed community type, not AFEs, was significantly associated with overweight/obesity status, suggesting children in suburban and especially rural/small town residences (odds ratio 5.11; 95% CI [1.59, 16.39]) could be priority populations for childhood obesity prevention programs.

**Conclusion:**

Findings of this multi-site study inform the feasibility of partnering with home visiting programs to reach and measure a diverse sample of children and families in need of childhood obesity prevention.

## Significance

*What is already known on this subject?* Home visiting programs are a recommended gateway for early childhood obesity prevention efforts, but the feasibility of implementing this recommendation is understudied.

*What this study adds?* This multi-site, multi-state study evaluated the feasibility of collecting anthropometric measurements through an established home visiting program, including the ability to reach children with overweight or obesity, from diverse backgrounds, and with adverse family experiences. This is an important step before implementing and scaling an early childhood obesity prevention program. Findings inform the development of childhood obesity prevention partnerships with home visiting programs.

## Introduction

### I: A Case for Action to Address Obesity

The obesity prevention framework (OPF) for translating evidence into action (Swinburn et al., 2005) defines five issues: (I) building a case for action; (II) identifying points of intervention; (III) defining opportunities for action; (IV) evaluating potential interventions; and (V) selecting specific policies, programs, and actions.

The case for addressing the U.S. obesity crisis is overwhelmingly clear given 74% of U.S. adults ≥ 20 years have overweight or obesity (Centers for Disease Control and Prevention & National Center for Health Statistics, 2021), and an estimated 86% will by 2030 (Hruby & Hu, 2015; Wang et al., 2008).

### II: Identifying Points of Intervention

Evidence supports addressing obesity in the earliest stages of life (Taveras, 2016). Healthy People 2030 set a goal of reducing the proportion of U.S. children and adolescents with obesity ages 2–19 years from 17.8% in 2013–2016 to 15.5% by 2030 (Healthy People, 2030). By 2017–2018, however, the proportion increased to 19.3%, with an additional 16.1% overweight (Fryar et al., 2020). There is no evidence of a decline in childhood obesity, rather there is a significant increase in obesity among children, including aged 2–5 years (Hu & Staiano, 2022).

### III: Defining Opportunities for Action

Childhood obesity prevention efforts have had limited success, especially among vulnerable populations, but home visiting programs reach families of young children at risk for obesity and offer potential for large-scale implementation (Salvy et al., 2017). Studies regarding the feasibility, acceptability, and efficacy of implementing early childhood obesity prevention content into home visitation programs are lacking (Zeldman et al., 2023).

Parents as Teachers (PAT) (https://parentsasteachers.org/) is an established, federally recognized evidence-based home visiting model with the potential to inform these efforts. PAT serves families with children prenatal through kindergarten via personal visits delivered primarily at home. PAT services include group connections, screenings and assessments with validated instruments, and referrals for basic needs such as housing and education. A family can enroll regardless of need or risk. PAT goals are to: (1) increase parent knowledge of early childhood development, (2) provide early detection of developmental delays and health issues, (3) prevent child abuse and neglect, and (4) increase children’s school readiness and success. In 2020–2021, PAT’s model was implemented across 966 affiliate sites in the U.S. and 4 other countries through 971,779 personal visits to 82,511 families and 101,173 children.

### IV: Evaluating Potential Interventions

We developed, implemented, and tested a proof-of-concept protocol for collecting child anthropometric data through PAT. We previously reported on the formative and process evaluation measures of that implementation, including the acceptability to stakeholders, percentage of data that met eligibility criteria, and percentage of measurement goals reached (Kapp et al., 2020). Guided by the RE-AIM framework, the objective of this study was to evaluate the *reach* of our proof-of-concept effort in measuring vulnerable children, i.e. with overweight or obesity, and with diversity of socioeconomic status (SES), ethnicity, locality, and gender. This informs the likelihood of an intervention reaching the known inequities in the distribution of obesity (Swinburn et al., 2005).

### V: Selecting Specific Policies, Programs, and Actions

We included an assessment of adverse family experiences (AFEs), given emerging evidence suggesting AFEs [and adverse childhood experiences (ACEs)] in childhood or adolescence are associated with obesity development (Gardner et al., 2019; Lynch et al., 2016; McKelvey et al., 2019). AFEs are a category of ACEs; ACEs traditionally include psychological abuse; physical abuse; and sexual abuse; as well as household dysfunction including substance abuse, mental illness, violence toward the mother, and other criminal behavior (Felitti et al., 1998; National Center for Injury Prevention and Control Division of Violence Prevention, 2019). An estimated 61% of U.S. adults experience ≥ 1 ACE category and 15.6% experience ≥ 4 categories (Merrick et al., 2019). ACEs increase the risk of obesity via chronic stress and coping behaviors such as overeating (Felitti et al., 1998; Merrick et al., 2019). To date, we know of no articles that have examined ACEs or AFEs and early childhood obesity in a home visiting setting, particularly with robust measurement of the family context. We hypothesize that AFEs are associated with early childhood obesity.

## Methods

### Site and Mother–Child Eligibility

We previously reported on our methodology and findings from the formative and process evaluation (Kapp et al., 2020), which is repeated here in brief. PAT’s National Center (PATNC) offers to sites a robust data management system nicknamed “Penelope” to track families’ services and outcomes. Site eligibility for our study required that sites: were using Penelope for ≥ 12 months; were accredited to implement the evidence-based model; met PATNC’s endorsement for quality; were implementing the Life Skills Progression (LSP) instrument (Wollesen, 2006); and had a robust number of eligible children. The LSP was developed to collect clinical and outcomes measures through home visiting. LSP has a reported construct validity ranging from α of 0.64–0.99 across scales, and interrater reliability estimated at 70–90%. LSP has a test–retest reliability reported at 0.90 for average inter-item correlation scores.

The four participating sites (a school district, federally qualified health center, and two non-profit organizations) were in South Carolina, Texas, Illinois, and Florida. Sites employed 5–50 home visitors. We shipped equipment to sites, and PATNC provided sites lists of eligible children. Training included measuring children 2–3 times for accuracy at a single visit. Sites received compensation for meeting measurement goals.

Child eligibility included: ages ≥ 6 months to < 5 years as of September 1, 2019, and active enrollment in PAT, with a completed child health record. Mother eligibility included a baseline LSP assessment.

Some sites incorporated measurements into the next scheduled home visit or group connections designed to bring families and children together to share information about parenting issues and child health and development. Sites recorded 260 child measurements, reaching 91% of their goals, over a 6-week period ending in November 2019.

### Child Anthropometric Measurement

For a child < 24 months, weight-for-length (WFL) is the recommended measure as compared to the World Health Organization (WHO) sex-specific growth standards. For children ages ≥ 24 months, body mass index (BMI) is recommended using the Centers for Disease Control and Prevention (CDC) growth charts. CDC’s SAS code calculates WFL and BMI measures and flags extreme values (Division of Nutrition; Physical Activity; and Obesity; National Center for Chronic Disease Prevention and Health Promotion, 2019a, 2019b). Our outcome is child anthropometric measurements as yes/no for overweight or obesity at the ≥ 85th (overweight) and ≥ 95th (obese) percentiles. We previously reported 214/260 records after exclusions and flagged data (Kapp et al., 2020). For this analysis the final sample included 248 children after 12 were excluded for being outside of the age eligibility criteria, and we were able to review and correct flagged data to centimeters instead of inches.

### Adverse Family Experiences (AFEs)

This analysis defines the presence of AFEs through the mother’s baseline LSP assessment (scores of 1–2 on scales suggest needs or dangerous conditions) and other PAT variables or assessments. PAT assessments are typically completed within 90–120 days of family enrollment. We label these as AFEs instead of ACEs given they reflect the child’s contextual environment and are not self-reported by the child.

**Psychological abuse** was identified from the LSP nurturing scale. **Physical abuse** was defined from the LSP discipline scale or PAT indication of child abuse or neglect reported or substantiated. **Substance abuse** was defined from the LSP substance use/abuse scale, or PAT indication of the parent’s persistent use of substances despite negative social, interpersonal, legal, medical, or other consequence at any point during the child’s lifetime (including prenatal). **Mental illness** in the home was defined from the mother’s LSP depression/suicide and mental illness scores; PAT indication of a parent with mental health issues (i.e., a thought, mood, or behavioral disorder associated with distress and/or impaired functioning determined by parent report or diagnosis); or the mother screened positive for depression (score of ≥ 13 on the Edinburgh Postnatal Depression Scale or ≥ 10 on the Patient Health Questionnaire-9) (Traube et al., 2022). **Mother has been treated violently** was identified from the LSP family/extended family scale; boyfriend, father of baby, or spouse scale; friends/peers scale; or PAT indication that the female parent is a survivor of intimate partner violence per self-report, positive screening, or court proceedings during the child’s lifetime. Family history of **criminal behavior** was identified by PAT questions indicating a parent was incarcerated in federal or state prison or local jail, halfway house, part of a boot camp or weekend program during the child’s lifetime, or a family member had been involved with the correctional system. Our AFEs index sums the child’s identified AFEs.

### Structural and Household Stressors

We created a “structural and household stressors” (SHS) index to sum additional stressors. Caregiver **low education** was identified from LSP < 12th grade education/education scales or PAT indicated a high school diploma or equivalency was not attained and the parent was not enrolled. **Low income** was defined from the LSP income scale or PAT indicated that the family was eligible for free and reduced lunches; public housing; childcare subsidy; Special Supplemental Nutrition Program for Women, Infants, and Children (WIC); food stamps/Supplemental Nutrition Assistance Program (SNAP); Temporary Assistance for Needy Families; Head Start/Early Head Start; and/or Medicaid. **Underemployment** was defined from the LSP employment scale or PAT indicated the mother as primary parent was not employed. **Immigration needs** were defined from the LSP immigration scale or PAT identified the family as a recent immigrant or refugee family (≥ 1 parents foreign born and entered the U.S. ≤ 5 years prior). **Unstable housing** was defined from the LSP housing scale or PAT identified the family as lacking a fixed, regular, or adequate nighttime residence, living in motels, residing in shelters or in public or private places not designated for sleeping; or the mother as primary parent was homeless and sharing housing, homeless in some other arrangement, or living in public housing. **Childcare needs** were identified from the LSP childcare scale. **Single parent** was identified by PAT. PAT identified a **young parent** as pregnant or parenting < age 21 years. PAT defined a **parent with a disability or chronic health condition** as having a physical or cognitive impairment that substantially limits the ability to parent. PAT defined a **child with a disability or chronic health condition** as a child with a significant delay, disability, or condition that affects developmental domains and/or overall family well-being.

### Other Definitions

**Community type** included major city (population ≥ 500,000) or urban (densely settled area with a population of ≥ 50,000); suburban (an identifiable community as part of a larger urban area); or small town (population ≥ 2500) or rural (population < 2500). **Follow-up time** was defined as years from the family’s PAT start to the child’s anthropometric measurement date.

### Data Analyses

Data were analyzed in SAS version 9.4 (SAS Institute Inc., Cary, NC). We characterized the study sample with frequencies and percentages, or medians and ranges, as appropriate. We tested the association between AFEs and early childhood overweight/obesity using multiple logistic regression; we calculated odds ratios and Wald confidence intervals, along with Type 3 Analysis of Effects *p* values (alpha = .05 for significance and .1 for trends). We reported the Hosmer–Lemeshow goodness-of-fit test for model specification, and the C-statistic for model discrimination. The University of Missouri Institutional Review Board reviewed and approved this study.

## Results

Children in this sample were followed by PAT for a median of 1.2 years, ranging from 0.09 to 4 years. This sample of children reflected: 50% female, 64.2% Hispanic/Latinx, 15.8% non-Hispanic Black, and 42.3% from rural/small towns (Table 1).

**Table 1:** Characteristics of 248 children in child anthropometric measurement proof-of-concept study

Variable	Overall
	n (%)^a^
Child’s age group	
6 to < 24 months	153 (61.7)
24 months to 5 years	95 (38.3)
Child’s age, median (range)	1.8 (0.5, 4.1)
Child’s sex	
Female	124 (50.0)
Male	124 (50.0)
Child’s race/ethnicity	
Hispanic/Latinx	154 (64.2)
Non-Hispanic Black	38 (15.8)
Non-Hispanic White	48 (20.0)
Family community type	
Suburban	85 (34.6)
Rural + Small town	104 (42.3)
Major city + Urban	57 (23.2)
Site’s state	
Florida	86 (34.7)
Illinois	63 (25.4)
South Carolina	40 (16.1)
Texas	59 (23.8)
Number of children in the household	
1	153 (62.2)
2	79 (32.1)
≥ 3	14 (5.7)
Follow-up time: years in the PAT program from the child’s PAT start date to child’s measurement, median (range)	1.20 (0.09, 3.98)
Family number of home visits, median (range)	37 (7–91)

^a^Unless otherwise indicated

Findings reveal 38.6% of the children < 24 months of age had overweight and 28.1% obesity, while 34.7% of the children ≥ 24 months had overweight and 22.1% obesity (Table 2). Overall, 37.1% of the children in the sample met or exceeded the respective age group standard for having overweight or obesity.

**Table 2:** Overweight and obesity outcomes of 248 children in proof-of-concept study

Age group	≥ 85th percentile category		≥ 95th percentile category	
	Yes	No	Yes	No
	n (%)	n (%)	n (%)	n (%)
< 24 months^a^	59 (38.6)	94 (61.4)	43 (28.1)	110 (71.9)
≥ 24 months^b^	33 (34.7)	62 (65.3)	21 (22.1)	74 (77.9)
Overall	92 (37.1)	156 (62.9)	64 (25.8)	184 (74.2)

^a^Calculated from World Health Organization (WHO) sex-specific growth standards

^b^Calculated from Centers for Disease Control and Prevention (CDC) growth charts

Children’s households had substantial SES needs: 73.8% had low education, 87.1% low income, and 59.3% underemployment. Overall, 89.1% of the children had ≥ 2 SHS, the most common being low income (Table 3).

**Table 3:** Distribution of structural and household stressors and adverse family experiences for 248 children in proof-of-concept study

Variable	Overall	Overweight or obese	
	n (%)	Yes	No
Structural and household stressors (SHS) (yes)			
Low education	183 (73.8)	72 (78.3)	111 (71.2)
Low income	216 (87.1)	84 (91.3)	132 (84.6)
Under employment	147 (59.3)	52 (56.5)	95 (60.9)
Immigration needs	81 (32.7)	30 (32.6)	51 (32.7)
Unstable housing	16 (6.5)	10 (10.9)	6 (3.9)
Under insured	73 (29.4)	24 (26.1)	49 (31.4)
Child care needs	94 (37.9)	33 (35.9)	61 (39.1)
Single parent	61 (24.7)	31 (34.1)	30 (19.2)
Young parent	41 (16.6)	18 (19.8)	23 (14.7)
Parent with a disability	18 (7.3)	3 (3.3)	15 (9.6)
Child with a disability	30 (12.2)	12 (13.2)	18 (11.5)
Sum SHS			
0–1	27 (10.9)	5 (5.4)	22 (14.1)
2–3	72 (29.0)	27 (29.4)	45 (28.9)
≥ 4	149 (60.1)	60 (65.2)	89 (57.1)
Adverse family experiences (AFEs) (yes)			
Psychological abuse	1 (0.4)	1 (1.1)	0 (0.0)
Physical abuse	12 (4.8)	5 (5.4)	7 (4.5)
Substance abuse	9 (3.6)	4 (4.4)	5 (3.2)
Mental illness	42 (16.9)	21 (22.8)	21 (13.5)
Mother treated violently	61 (24.6)	25 (27.2)	36 (23.1)
Family criminal behavior	13 (5.2)	5 (5.4)	8 (5.1)
Sum AFEs			
0	153 (61.7)	51 (55.4)	102 (65.4)
1	66 (26.6)	26 (28.3)	40 (25.6)
≥ 2	29 (11.7)	15 (16.3)	14 (9.0)

Regarding AFEs, 38.3% of the children had ≥ 1; the most common being that 24.6% of children’s mothers had been treated violently.

Results of the multivariable model (Table 4) adjusting for child age, sex, race/ethnicity, community type, number of children in the household, SHS, site, number of home visits and follow-up time did not indicate a significant relationship between AFEs and overweight/obesity. Community type was significant, suggesting children in suburban and especially in rural/small town residences are at greater risk for overweight/obesity compared with children in major city/urban residences.

**Table 4:** Final model predicting overweight or obesity among 248 children in a proof-of-concept study

Variable	Odds Ratio (95% CI)^a^	*p* value
Child’s age (years)	0.61 (0.36, 1.05)	0.08
Child’s sex		
Female	0.69 (0.39, 1.22)	0.21
Male	Referent	
Child’s race/ethnicity		
Hispanic/Latinx	1.21 (0.50, 2.88)	0.90
Non-Hispanic Black	1.03 (0.37, 2.91)	
Non-Hispanic White	Referent	
Family community type		
Suburban	3.24 (1.03, 10.18)	0.02
Rural + Small Town	5.11 (1.59, 16.39)	
Major City + Urban	Referent	
Number of children in the household	1.17 (0.68, 2.02)	0.57
Sum SHS		
0–1	0.30 (0.08, 1.10)	0.18
2–3	0.97 (0.51, 1.85)	
≥ 4	Referent	
Sum AFEs		
0	Referent	0.71
1	1.01 (0.52, 1.96)	
≥ 2	1.47 (0.57, 3.78)	

Included adjustment for project site, number of home visits, follow-up time

^a^Full model: Hosmer–Lemeshow Goodness of Fit: 0.98; C-Statistic: 0.69

## Discussion

This study addresses a significant gap in the literature and provides supporting evidence that home visiting programs are a promising gateway for early childhood obesity prevention efforts. That 37.1% of the children in this sample already have overweight or obesity before the age of 5 is alarming. Results also indicate that home visiting partnerships can reach children with many of the known inequities in the distribution of obesity (Swinburn et al., 2005). Our sample of children included 50% female, 64.2% Hispanic/Latinx, 15.8% non-Hispanic Black, and 87.1% low income, with 42.3% from rural/small town localities. That participating children were enrolled in PAT for up to 4 years with a median of 37 home visits is promising for developing obesity prevention efforts that offer sustained contact and support for behavior change.

Our findings inform the OPF’s guidance for translating evidence into action. Rural/small-town settings were associated with early childhood obesity after robust adjustment for child, parent, and family characteristics, suggesting a priority area for intervention. This finding is consistent with reports that adults in rural areas are more likely to be obese (Befort et al., 2012; Patterson et al., 2004). Given the limited sample size for large-scale inference, we consider the findings preliminary, and worthy of revisiting with a scaled intervention.

Family members, as the primary source of a child’s experiences, influence a child’s environment the most (Maggi et al., 2005; Siddiqi et al., 2007). In our study, AFEs were not significantly associated with overweight/obesity. It is possible, however, that indirect influences on overweight/obesity are less identifiable at this age (< 5 years). Post-hoc contingency tables suggest a relationship between AFEs and early childhood obesity, with 33.3%, 39.4%, and 51.7% of children with 0, 1, and ≥ 2 AFEs, respectively, having overweight/obesity. This relationship deserves further investigation as AFEs have not been adequately studied in early childhood.

Limitations include that findings are not necessarily generalizable to all PAT sites or U.S. children. This is a proof-of-concept study and does not reflect a sample size indicative of a fully scaled intervention. Strengths of this study include that the settings reflect real-world family environments to inform effectiveness trials, and we achieved a diverse, high-needs sample from multiple sites and four states.

This work uniquely contributes to the literature gap regarding the feasibility of implementing early childhood obesity prevention efforts into home visitation programs.

## Data Availability

Contact allison.kemner@parentsasteachers.org and kappj@health.missouri.edu.

## Code Availability

Contact kappj@health.missouri.edu.

## References

[CR1] Befort CA, Nazir N, Perri MG (2012). Prevalence of obesity among adults from rural and urban areas of the United States: Findings from NHANES (2005–2008). The Journal of Rural Health.

[CR2] Centers for Disease Control and Prevention, & National Center for Health Statistics. (2021). *Obesity and Overweight*. Retrieved July 8, 2021, from https://www.cdc.gov/nchs/fastats/obesity-overweight.htm

[CR3] Division of Nutrition; Physical Activity; and Obesity; National Center for Chronic Disease Prevention and Health Promotion. (2019a). *A SAS Program for the 2000 CDC Growth Charts (ages 0 to <20 years)*. Centers for Disease Control and Prevention. Retrieved August 5, 2019, from https://www.cdc.gov/nccdphp/dnpao/growthcharts/resources/sas.htm

[CR4] Division of Nutrition; Physical Activity; and Obesity; National Center for Chronic Disease Prevention and Health Promotion. (2019b). *A SAS Program for the WHO Growth Charts (ages 0 to <2 years)*. Centers for Disease Control and Prevention. Retrieved July 16, 2019, from https://www.cdc.gov/nccdphp/dnpao/growthcharts/resources/sas-who.htm

[CR5] Felitti VJ, Anda RF, Nordenberg D, Williamson DF, Spitz AM, Edwards V, Koss MP, Marks JS (1998). Relationship of childhood abuse and household dysfunction to many of the leading causes of death in adults. The Adverse Childhood Experiences (ACE) Study. American Journal of Preventice Medicine.

[CR6] Fryar, C. D., Carroll, M. D., & Afful, J. (2020). *Prevalence of overweight, obesity, and severe obesity among children and adolescents aged 2–19 years: United States, 1963–1965 through 2017–2018*. National Center for Health Statistics Health E-Stats. https://www.cdc.gov/nchs/data/hestat/obesity-child-17-18/overweight-obesity-child-H.pdf 41779889

[CR7] Gardner R, Feely A, Layte R, Williams J, McGavock J (2019). Adverse childhood experiences are associated with an increased risk of obesity in early adolescence: A population-based prospective cohort study. Pediatric Research.

[CR8] Healthy People 2030. (2021). *Reduce the proportion of children and adolescents with obesity—NWS‑04*. Office of Disease Prevention and Health Promotion, Office of the Assistant Secretary for Health, Office of the Secretary, U.S. Department of Health and Human Services. Retrieved July 8, 2021, from https://health.gov/healthypeople/objectives-and-data/browse-objectives/overweight-and-obesity/reduce-proportion-children-and-adolescents-obesity-nws-04

[CR9] Hruby A, Hu FB (2015). The epidemiology of obesity: A big picture. PharmacoEconomics.

[CR10] Hu K, Staiano AE (2022). Trends in obesity prevalence among children and adolescents aged 2 to 19 years in the US from 2011 to 2020. JAMA Pediatrics.

[CR500] Kapp JM, Hall B, Kemner A (2020). Collecting early childhood obesity measurements through a home visiting program: A proof-of-concept study. Preventing Chronic Dissease.

[CR11] Lynch BA, Agunwamba A, Wilson PM, Kumar S, Jacobson RM, Phelan S, Cristiani V, Fan C, Finney Rutten LJ (2016). Adverse family experiences and obesity in children and adolescents in the United States. Preventive Medicine.

[CR12] Maggi, S., Irwin, L. G., Siddiqi, A., Poureslami, I., Hertzman, E., & Hertzman, C. (2005). *Knowledge network for early child development. Analytic and strategic review paper: International perspectives on early child development*. World Health Organization’s Commission on the Social Determinants of Health. http://www.who.int/social_determinants/resources/ecd.pdf?ua=1

[CR13] McKelvey LM, Saccente JE, Swindle TM (2019). Adverse childhood experiences in infancy and toddlerhood predict Obesity and health outcomes in middle childhood. Childhood Obesity.

[CR14] Merrick MT, Ford DC, Ports KA, Guinn AS, Chen J, Klevens J, Metzler M, Jones CM, Simon TR, Daniel VM, Ottley P, Mercy JA (2019). Vital signs: Estimated proportion of adult health problems attributable to adverse childhood experiences and implications for prevention—25 States, 2015–2017. MMWR Morbidity Mortality Weekly Report.

[CR15] National Center for Injury Prevention and Control Division of Violence Prevention. (2019). *About the CDC-Kaiser ACE study*. Retrieved January 8, 2019, from https://www.cdc.gov/violenceprevention/childabuseandneglect/acestudy/about.html

[CR16] Patterson PD, Moore CG, Probst JC, Shinogle JA (2004). Obesity and physical inactivity in rural America. The Journal of Rural Health.

[CR17] Salvy SJ, de la Haye K, Galama T, Goran MI (2017). Home visitation programs: An untapped opportunity for the delivery of early childhood obesity prevention. Obesity Reviews.

[CR18] Siddiqi, A., Irwin, L. G., & Hertzman, C. (2007). *Total environment assessment model for early child development*. World Health Organization’s Commission on the Social Determinants of Health. http://www.who.int/social_determinants/resources/ecd_kn_evidence_report_2007.pdf?ua=1

[CR19] Swinburn B, Gill T, Kumanyika S (2005). Obesity prevention: A proposed framework for translating evidence into action. Obesity Reviews.

[CR20] Taveras EM (2016). Childhood obesity risk and prevention: Shining a lens on the first 1000 days. Childhood Obesity.

[CR21] Traube DE, Molina AP, YingWangKay S, Kemner A (2022). Perinatal mental health support and early childhood home visitation during COVID-19. Prevention Science.

[CR22] Wang Y, Beydoun MA, Liang L, Caballero B, Kumanyika SK (2008). Will all Americans become overweight or obese? estimating the progression and cost of the US obesity epidemic. Obesity.

[CR23] Wollesen L, Peifer K (2006). Life skills progression. An outcome and intervention planning instrument for use with families at risk.

[CR24] Zeldman J, Varela EG, Gorin AA, Gans KM, Gurka MJ, Bernier AV, Mobley AR (2023). Home visitation program staff attitudes and intentions towards using digital technology to educate families about preventing early childhood obesity: A qualitative study. Matern and Child Health Journal.

